# Towards a better understanding of real-world home-visiting programs: a large-scale effectiveness study of parenting mechanisms in Brazil

**DOI:** 10.1136/bmjgh-2023-013787

**Published:** 2024-02-20

**Authors:** Morgan Rebecca Healy, Eduardo Viegas da Silva, Anton Rask Lundborg, Fernando Pires Hartwig, Tiago Neuenfeld Munhoz, Adriane Xavier Arteche, Paul G Ramchandani, Joseph Murray

**Affiliations:** 1 PEDAL, Faculty of Education, University of Cambridge, Cambridge, UK; 2 Postgraduate Program in Epidemiology, Federal University of Pelotas, Pelotas, Brazil; 3 Human Development and Violence Research Centre (DOVE), Federal University of Pelotas, Pelotas, Brazil; 4 University of Copenhagen Department of Mathematical Sciences, Kobenhavn, Denmark; 5 Department of Psychology, Federal University of Pelotas, Pelotas, Brazil; 6 Department of Psychology, Pontifical Catholic University of Rio Grande do Sul, Porto Alegre, Brazil

**Keywords:** Child health, Health policy, Epidemiology, Maternal health, Prevention strategies

## Abstract

**Background:**

The scale-up of parenting programmes to support early childhood development (ECD) is poorly understood. Little is known about how and when early interventions are most effective. Sustainability of ECD programming requires a better understanding of the mechanisms of real-world interventions. We examined the effects on caregiving practices of Primeira Infância Melhor (PIM), a state-wide home-visiting programme in Brazil.

**Methods:**

This propensity score matched, longitudinal, quasiexperimental study uses data from the 2015 Pelotas Birth Cohort. We matched children who received PIM at any age with other cohort children on 25 key covariates. Sensitivity, guidance and responsiveness were assessed using video-recorded play tasks. Coerciveness and the parent–child relationship were assessed using the Parenting and Family Adjustment Scales. All parenting outcomes were examined at age 4 years. Separate moderation analyses were conducted for each effect modifier: family income, child age and duration of participation.

**Results:**

Out of 4275 children in the cohort, 797 were enrolled in PIM up to age 4 years. 3018 children (70.6%) were included in the analytic sample, of whom 587 received PIM and 2431 were potential controls. We found a positive effect of PIM on responsiveness (β=0.08, 95% CIs 0.002 to 0.16) and sensitivity (β=0.10, 95% CIs 0.02 to 0.19). No effect was found for any secondary outcomes. Moderation analyses revealed a stronger positive effect on sensitivity for low-income parents (β=0.18, 95% CIs 0.03 to 0.34).

**Conclusion:**

A state-wide, home-visiting programme in Brazil improved aspects of responsive caregiving. Effects were more pronounced for low-income families, suggesting benefits of purposeful targeting.

**WHAT IS ALREADY KNOWN ON THIS TOPIC**

- Parenting interventions have the potential to improve child development via multiple parent-related pathways and can attenuate the effects of poverty on infant development.
- Effects of these interventions have been found to diminish when delivered at-scale in real-world settings.
- A state-wide, population-based home-visiting programme (Primeira Infância Melhor (PIM)) in Southern Brazil had some benefits for child development.

**WHAT THIS STUDY ADDS**

- The PIM home-visiting programme in Southern Brazil was found to improve sensitive and responsive caregiving, a key mechanistic parenting pathway.
- Effects on parent sensitivity were greater for low-income caregivers, suggesting the potential of such programmes to reduce risks associated with economic deprivation.

**HOW THIS STUDY MIGHT AFFECT RESEARCH, PRACTICE OR POLICY**

- Home-visiting public policy targeting nurturing care can improve responsive caregiving, a key protective factor of child development, in standard-care, large-scale implementation settings.
- Future research is needed to further explore whether and by what means large-scale parenting programmes are differentially effective at improving parenting across varying implementation conditions and income subgroups.

## Introduction

### Early parenting in low-resource settings

The first 1000 days of a child’s life lay the foundation for human capital development.^1^ Brain architecture is especially sensitive during this early period and is heavily shaped by relational and environmental conditions.^2^ Early competencies in cognition and emotional regulation formed during infancy consistently predict later educational attainment, job productivity and mental health.^3 4^ Despite widespread scientific evidence and public recognition of early childhood development (ECD), globally 39% of children are at risk of not achieving their developmental potential. This is estimated to result in a future income loss of 20% each year.^5^

Socioeconomic deprivation is one mechanism through which deficits in ECD emerge and are perpetuated.^6^ Consistent evidence over the past 20 years has revealed poverty’s long-term biobehavioural and epigenetic effects on brain functioning^7^ and its cascading effect across generations.^8^ Poverty and associated risk factors adversely impact parents and their caregiving practices.^5^ Parents living in poverty are less likely to read or play with their children and have fewer developmentally appropriate play materials at home.^8^ Recent meta-analytic research suggests that children in low-income and middle-income countries (LMICs) are six times less likely than children in high-income countries (HICs) to experience high stimulation at home and access early childhood education (9.2% vs 60.7%).^5^ With over 90% of children under age 5 living in LMICs, it is a major challenge to provide adequate support for optimal development, leaving no child behind.^9^

Quality stimulation during the early childhood period can attenuate the detrimental effects of poverty on brain architecture and stress reactivity.^10 11^ The extent to which caregivers are responsive and reciprocal in their interactions with infants and toddlers are key determinants of optimal child development.^12^ A substantial body of causal evidence has confirmed that early interventions which target nurturing parenting behaviours, particularly sensitive responsiveness, can lead to significant improvements in children’s cognitive, socioemotional and behavioural outcomes in the short and long term (for review see: Jeong *et al*,^13^ Aboud and Yousafzai^14^ and Sweet and Appelbaum^15^). Responsive parenting can also serve as a robust protective factor against poverty-related adverse outcomes.^16^

### Evidence from large-scale parenting programs in Latin America

Despite expansion in the scale of parenting programmes across the globe in recent years, reach has not been consistently met with impact. Many scaled interventions have failed to replicate,^17^ with null effects reported twice as often as efficacy trials.^18^

Another key uncertainty relates to the mechanisms by which parenting programmes improve child outcomes. Recently, policy-makers and ECD stakeholders at global levels have recommended moving away from ‘black box’ studies which assess impact on child outcomes alone^19^ towards seeking to understand the ‘agents of change’ and core components in the interventions themselves.^20^ Successful replication and scale-up of parenting programmes requires a better understanding of the key mechanisms in these early interventions in usual-care, real-world settings—specifically *how* they improve child development.^21^ Despite acknowledgement of their importance, few evaluations have actually assessed parenting mechanisms.^14^ A recent global meta-analysis, for example, reported that 40% of parenting intervention studies did not report on any parenting outcome.^13^

Across Latin America, only a handful of large-scale parenting programme evaluations have assessed intervention effects on caregiving. First, in Colombia, a weekly psychosocial stimulation programme based on ReachUp was tested across 96 municipal sites. Parenting practices were evaluated using UNICEF’s Multiple Cluster Family Care Indicator (MCI), a retrospective parent-self report measure of home stimulation. Improvements in the frequency of parent–child play were reported immediately post intervention.^22^ Second, in Peru, an evaluation of the Cuna Mas home-visting programme across 60 districts using the same measure resulted in a reduction of parents’ corporal and verbal punishment (6%–7%), while no effects on responsive parenting were reported.^12^ Third, in Mexico, a cluster randomised controlled trial (RCT) of the Educacion Inicial home-visting programme also using MICs, found a 13% increase in the number of play activities that parents engaged in with their children.^23^ Collectively, these studies suggest that large-scale parenting programmes have the potential to improve the frequency of some caregiving practices. However, their implementation by the authoring research team^22^ and exclusive reliance on parent self-report or summed counts of complex parent behaviours^12 23^ limit their capacity to clearly elucidate the behavioural parenting mechanisms by which these large-scale programmes operate.

Furthermore, there is also a dearth of real-world effectiveness studies in LMICs which assess heterogeneity of treatment effects by implementation features or meaningful subgroups. More research is needed to understand whether variation in population risk profiles may influence intervention effectiveness on parenting outcomes.^13^ The current study contributes to this literature by evaluating whether *Primeira Infância Melhor* (PIM), a state-wide, pragmatic home-visiting programme in Southern Brazil (1) improved five caregiving practices at 4 year follow-up and (2) whether effects were moderated by child age, duration of participation or family income. PIM was previously found to benefit child development at age 4 years, when implemented from gestation onwards.^24^

## Methods

We conducted a preregistered, propensity score matched, longitudinal, quasiexperimental study, using data from the 2015 Pelotas (Brazil) Birth Cohort. Pelotas is a medium-sized city, population 344 000, in southern Brazil. Overall, 42% of children 0–14 in Pelotas live in poverty compared with 34% across the state.^25^ Families were eligible for inclusion in the birth cohort if children were hospital delivered, residing within the urban boundaries of the city, and were born between 1 January 2015 and 31 December 2015. The cohort includes 4275 children, representing 99.1% of all children born within the city. Assessments were conducted at birth, 3, 12, 24 months and 4 years at families’ homes, the hospital or the university research centre.^26^

Primary data from the 2015 Pelotas birth cohort were linked with PIM state database based on the child’s name, mother’s name and child’s DOB in order to evaluate the effects of PIM on participants in the cohort. PIM funding is contingent on the number of children registered per city, so it is unlikely families received the programme without being registered.^24^

### Intervention

PIM is a state-wide home-visiting programme in Rio Grande do Sul, Brazil, which has been in operation since 2003 and reached 200 000 families to date.^27^ The programme targets socioeconomically vulnerable families, of which it currently reaches approximately 50% across the state (PIM, 2023). PIM became a public policy by law in 2006 and served as a model for Brazil’s federal parenting programme, Crianca Feliz (CF), which is the largest home-visiting programme in the world.^27^ Previous studies have found that children who received PIM demonstrated small reductions in infant mortality^28^ and school-reported behaviour problems^29^ and improvements in cognitive development.^24^ This is the first peer-reviewed evaluation of its kind to assess PIM’s effect on parent behavioural mechanisms.

The aim of PIM is to promote child development through the encouragement of responsive, play-based parent–child interactions and facilitate family uptake of social services. Each weekly visit centres around reviewing the previous weeks’ activities and introducing a new play activity to the parent–child pair.

PIM is conceptualised as one governmental strategy to reach socially vulnerable families that may not have access to the early education services.^30^ The programme is implemented by the state advising team and municipal coordinating teams. Supervisors work under the directive of the municipal team are assigned eight visitors to oversee. Visitors complete a 60-hour training and visit, at maximum, 20 families per week.^27^ No comprehensive curriculum is available to visitors. Instead, they are expected to plan for each visit by drawing from a guidebook of core activities or creating their own activity plan, which they discuss with their supervisors.^27^ Additional details about home visitor demographics and programme history can be found elsewhere.^24 27^

In the current study, 797 families of the 4275 families in the Pelotas birth cohort received PIM at any time between gestation and child age 4. Though PIM aims to target families with greater social vulnerability, no explicit selection criteria were used. Families included in the programme were identified by visitors during their daily work in low-income neighbourhoods, indicated by child services, or recommended by other families. Approximately two-thirds of families that received PIM belonged to the bottom two income quintiles, compared with a third of families from the cohort that did not receive PIM. Families receiving PIM also reported lower rates of maternal and paternal education and higher rates of depression and neighbourhood violence, known risk factors for poor child development, suggesting that the programme was successful at reaching a proportion of vulnerable families (online supplemental table 1).

10.1136/bmjgh-2023-013787.supp1 Supplementary data

### Outcomes

#### Primary outcomes

All parent outcomes were assessed at 4 years. For the three observational measures of parenting—responsiveness, sensitivity and guidance—approximately 400 of the assessments (10%) were double coded by graduate researchers who were blind to treatment allocation to ensure quality control.

Two directly observed measures of parent sensitive responsiveness were prespecified as the primary outcomes, given their theoretical alignment with PIM content and robust evidence base.^13 31^ Sensitive responsiveness refers to how accurately a parent notices their child’s signals and how promptly and appropriately they respond to them.^32^ Parent responsiveness was assessed using Responsive Interactions for Learning (RIFL),^33^ which is comprised of three observationally coded subscales: communicative clarity, mind-reading and mutuality building, yielding a composite score out of 11. A brief 5 min joint parent–child building task was coded for responsiveness. RIFL was recently validated in Brazil using CFA and demonstrated excellent internal consistency (α=0.94), with item-total correlations ranging from 0.61 to 0.88.^34^ Intraclass correlation scores (ICC) indicated moderate reliability^35^ (n=415; ICC=0.62).

Parent sensitive responsiveness was also assessed by an observational measure. The measure was previously used in South Africa and designed for young children.^36 37^ Caregivers were instructed to read a picture book with their child for 5 min. Higher scores on the 5-point Likert scale indicate higher rates of responsiveness. Intraobserver agreement on the measure was excellent (n=415; 99.4%; Kappa: 0.97).

#### Secondary outcomes

##### Parent guidance

Parent guidance was assessed via observation of the filmed don’t touch task and based on a coding scheme previously used in South Africa with children of a similar age group.^38 39^ For the task, the caregiver and child were presented with a box of interesting toys and instructed that they were not allowed to touch the toys until the experimenter returned. Parent verbal and physical guidance were ranked separately from 0 to 3 for each 20 s block and then averaged to produce a total score. Intraobserver agreement on the measure indicated excellent reliability (n=497; ICC=0.99)

##### Parent coerciveness and parent–child relationship

We measured coercive parenting and the quality of the parent–child relationship using the Parenting subscale of the Parenting and Family Adjustment Scales (PAFAS).^40^ PAFAS is a parent-self report measure which has been used previously in Brazil.^41^ A recent confirmatory factor analysis of the PAFAS Parenting subscale was applied at 4-year follow-up to the 2015 Pelotas cohort (n=3970). The validated scale used in the current study includes 14 of the original 18 items and showed a good reliability coefficient (0.91). The parental coerciveness subscale includes 4 of the original 5 items, with 12 points possible (online supplemental table 2). The parent–child relationship subscale includes all 5 original items, with 15 points possible (online supplemental table 2).

10.1136/bmjgh-2023-013787.supp2 Supplementary data

### Statistical analysis

Analysis plans were preregistered on the Open Science Framework(OSF; https://osf.io/2y3vn/). After linking the cohort and PIM databases, propensity scores were calculated for the probability of receiving PIM, conditional on baseline covariates. Analyses were conducted in R V.4.1.0. In logistic regression, 25 covariates were used to calculate the propensity scores, with participating in PIM as the dependent variable. The details about each covariate can be found in online supplemental table 3. All covariates were measured from maternal reports during the perinatal assessment at the hospital except the following: main caregiver until the child reached 3 months of age; maternal depressive symptoms and the couple’s relationship quality, which were measured at the 3-month assessment; childcare attendance, which was measured at the 2-year assessment; and neighbourhood violence, which was measured at the 4-year assessment.

10.1136/bmjgh-2023-013787.supp3 Supplementary data

We matched those who had received PIM (n=587) with remaining participants from the cohort who had not received PIM (n=2431) based on their propensity score. Participants were excluded from the analysis if they were missing data on any covariate or outcome variable given the small number of losses and difficulty of operationalizing imputation methods in analysis with double adjustment. The covariate with the highest percentage of missing data (couple’s relationship quality: 17.5%) was not included in the propensity score calculation, to minimise losses, but was checked during balance assessments.

Prior to running the logistic regression, initial balance was checked on each covariate to determine whether estimates were above the absolute 0.1 standardised mean difference (SMD) cut-off between the potential control and treatment groups.^42 43^ Those covariates which met SMD balance criteria prior to running the regression were excluded from the initial propensity score model, but were included in the balance assessment after matching. Covariates which were found unbalanced after initial matching were entered into a new logistic regression model until balance was achieved. Covariates were considered balanced after matching, if SMDs were below 0.1 across each covariate level and variance ratios (VRs) were >0.5 and <2, which are cut-off points found by previous researchers to denote considerable differences between two groups.^42 44^

Various matching approaches were tested, including nearest neighbour, optimal matching and full matching. Full matching was found to produce the most well-balanced pairings, with the smallest SMD differences and VRs across each covariate level. Full matching is a form of matching wherein all treatment and control units are allocated to a subclass and weighted. It is recognised as a combination of distance and stratum matching.^45^ Unlike 1:1 matching, full matching produces matching weights which are computed to produce an effective sample size and applied to the matched sample in the outcome analysis.^46^ The advantages of full matching are that no matching order is required to be prespecified—which can influence the quality of matching—and control units can be reused—which is particularly important when treated individuals differ markedly across key covariates from potential controls.^46 47^

Covariates which were found to be unbalanced after multiple matching specifications were re-entered into the outcome regression for double adjustment.^48^ For SE estimation, we used cluster robust errors to account for potential dependence between matched pairs.^49–51^ We applied bootstrapping (B=2999) to estimate the standard errors of the binary outcome, parent–child relationship, when covariates were re-entered into the logistic regression outcome model, with bias-corrected and accelerated CIs.^45 52^

All parenting outcomes remained untransformed in their original form, except the quality of the parent–child relationship, which was dichotomised. Analyses of our primary outcomes—parent responsiveness and sensitivity—were based on linear regression. Regarding secondary outcomes, parent guidance and coerciveness, were analysed by linear regression, while the quality of the parent–child relationship was analysed by logistic regression for direct estimation of the OR.

We first analysed the effect of PIM on parenting outcomes for families who enrolled at any time between pregnancy and child aged 4 years (n=3018). Next, moderation analyses were conducted. The intervention group was stratified for each of the three potential moderators—(1) low-income (bottom income tercile) versus high-income family (upper two income terciles); (2) joining PIM before versus after birth; (3) and receiving PIM for 12-months < versus ≥ 12 months. Full matching, using the steps outlined above, was conducted separately for each stratified subgroup drawing from the control group, which was randomly split in half to prevent overlap between the two matched sets. Following this, separate effects were estimated and Cochrane’s Q heterogeneity χ^2^ test was used to examine potential effect modification across the three moderators.

Of note, for the stratum which enrolled in PIM during pregnancy, 15 covariates were selected. This is because 11 of the original 25 covariates are plausible mediators of the effect of PIM starting during pregnancy on parenting outcomes. For the stratum which enrolled after birth and moderation questions related to family income and length of programme involvement, the same original 25 confounders were also used.

### Patient and public involvement

The public was not involved in the design or conduct of our research, given the intervention was nested in a birth cohort study and used secondary data from the PIM data system. The municipal and state managers of PIM were involved in the planning of this evaluation from the beginning, including in the selection of relevant parenting outcomes. The study’s high follow-up rate reflects its exceptional communication with families and positive reception within the Pelotas community. The results are being disseminated and discussed with those responsible for implementing the programme, to improve its impact.

## Results

### Main analyses

**Figure 1:**
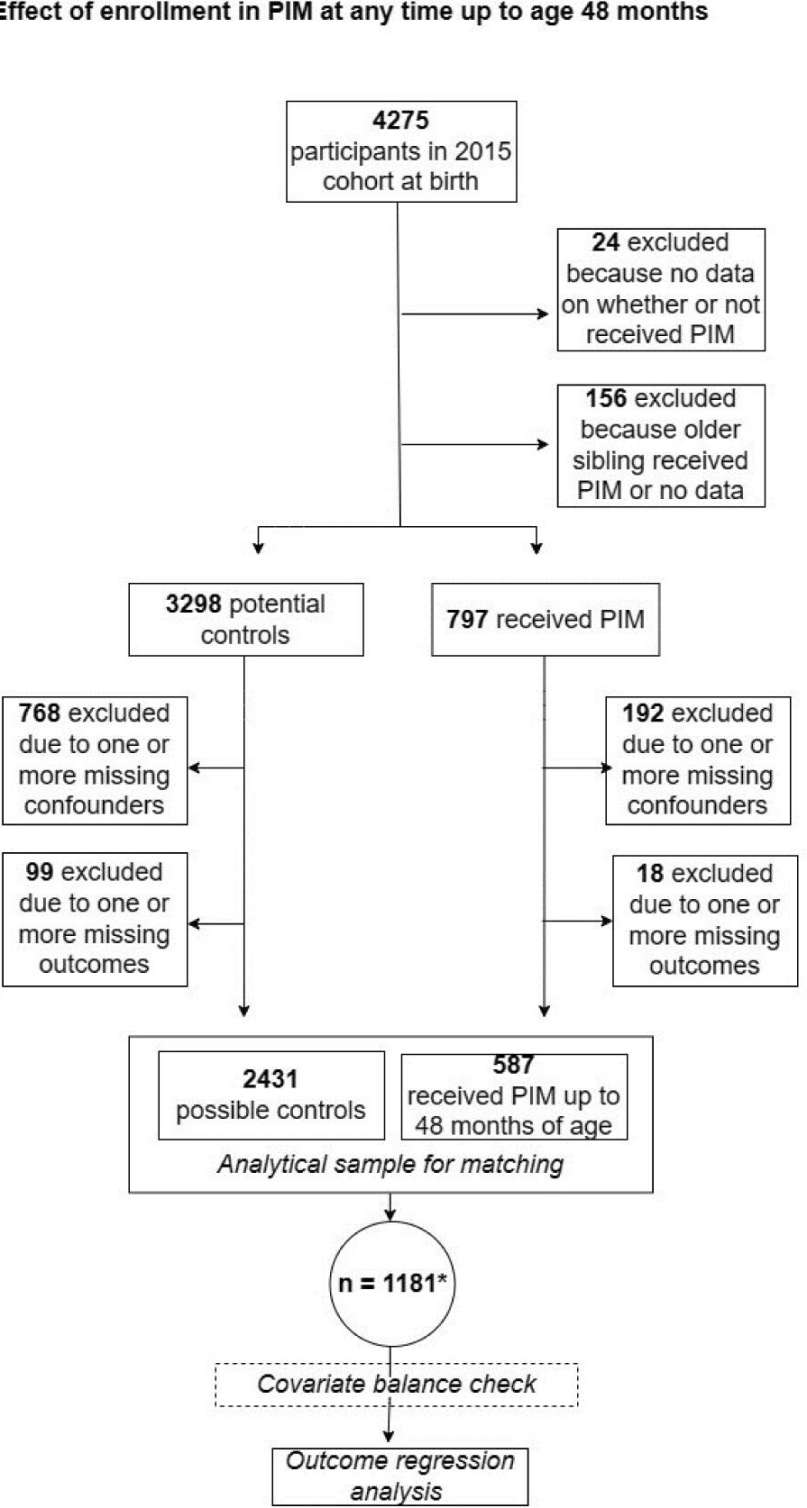
Flow chart showing the number of children for whom propensity scores were calculated in the primary matched analyses and reasons for covariate and outcome exclusion. PIM, Primeira Infância Melhor.

Out of 4275 children in the Pelotas cohort, 797 were enrolled in PIM at any point up to 4 years of age. 3018 children (70.6%) were included in the analytic sample, of whom 587 were enrolled in PIM and 2431 were potential controls. Most exclusions from the original sample were due to missing data on key covariate or outcome variables (figure 1).

Online supplemental figure 1 presents two love plots which compare the SMDs and VRs between families who received PIM at any time versus potential controls, before versus after matching on the 25 confounders (pink vs blue dots). The dotted line indicates threshold cut-offs of 0.1 for SMD and 0.5 and 2 for VRs. Full matching resulted in matches well-below the SMD and VR thresholds for all covariates.

10.1136/bmjgh-2023-013787.supp4 Supplementary data

Comparing families who received PIM with matched controls, we found a positive effect of PIM on parent responsiveness (β=0.08, 95% CIs 0.002 to 0.16, d=0.10, p=0.04) and parent sensitivity (β=0.10, 95% CIs 0.02 to 0.19, d=0.13, p=0.02) (table 1). No strong evidence for an effect was found for coerciveness, guidance or the parent–child relationship.

**Table 1:** Effects of any enrolment in Primeira Infância Melhor up to 4 years on parenting outcomes

Full matched analysis (n=1181*)				
Outcome	β	95% CI**	P value	Cohen’s d
Linear regression for mean parenting score (β)				
Parent responsiveness	0.08	0.002 to 0.16	0.04†	0.10
Parent sensitivity	0.10	0.02 to 0.19	0.02†	0.13
Parent coerciveness	0.002	−0.04 to 0.06	0.98	0.001
Parent guidance	0.01	−0.04 to 0.06	0.63	0.03
Logistic regression for OR				
	**OR**	95% CI*	**P value**	
Quality of parent–child relationship	1.07	0.85 to 1.33	0.56	

*Effective sample size (ESS) is the effective sample size and is used in cases of full matching when matched samples are calculated via stratification and weighting. In the case of this analysis, in the ESS, n=1181, where there were 594 controls and 587 treated individuals.

†, Denotes that p value is below 0.05 threshold.

### Moderation analyses

First, effects of PIM were estimated comparing families in the bottom income tercile to those in the upper two terciles (online supplemental figure 2). For higher income families, matching yielded covariate balance well within all SMD and VR thresholds (online supplemental figure 3). For the 313 low-income families who received PIM, matching resulted in good balance for most but not all covariates (online supplemental figure 4). No evidence of an effect was found for any parenting outcome in the higher income group. A positive effect on parent sensitivity (β=0.18, 95% CIs 0.03 to 0.34, d=0.22, p=0.02) was found for low-income parents. Cochrane’s Q heterogeneity test which compared the sensitivity scores of the low and relative higher-income groups produced a p value of 0.11, just above the cut-off criteria (table 2).

10.1136/bmjgh-2023-013787.supp5 Supplementary data

10.1136/bmjgh-2023-013787.supp6 Supplementary data

10.1136/bmjgh-2023-013787.supp7 Supplementary data

**Table 2:** Effects of receiving PIM on low-income caregivers’ or higher-income caregivers’ parenting outcomes

Outcome	Low-income caregivers (n=436*)			Higher-income caregivers (n=556*)			
	β	95% CI	P value	β	95% CI	P value	Heterogeneity P values
Linear regression for mean parenting score (β)							
Parent responsiveness	0.01	−0.14 to 0.16	0.89	0.09	−0.04 to 0.22	0.19	0.45
Parent sensitivity	0.18	0.03 to 0.34	0.02†	0.02	−0.11 to 0.15	0.78	0.11
Parent coerciveness	0.02	−0.34 to 0.39	0.89	0.11	−0.24 to 0.47	0.53	0.73
Parent guidance	−0.01	−0.07 to 0.06	0.87	−0.01	−0.08 to 0.06	0.69	0.87
Logistic regression for OR estimated via regression model (high-income caregivers) or bootstrapping (n=2999) using BCa (low-income caregivers)							
	**OR**	**95% CI**		**OR**	**95% CI**	**P value**	
Quality of parent–child relationship	0.89	0.44 to 1.21		0.87	0.62 to 1.21	0.40	0.92

*Effective sample size (ESS) is the effective sample size and is used in cases of full matching when matched samples are calculated via stratification and weighting. In the case of this moderation analyses, the ESS for low-income families is n=436, where there are 123 controls and 313 treated individuals. The ESS for higher income families is n=556, where there are 282 controls and 274 treated individuals.

†, Denotes p value below the 0.05 threshold; BCa, Bias-corrected and accelerated bootstrap interval.

Next, we estimated the effects of PIM for those families who received the programme during pregnancy compared with after birth (online supplemental figure 5). The two strata demonstrated high-quality covariate balance well below cut-offs on all SMDs and VRs (online supplemental figures 6 and 7). We found no strong statistical evidence for effect modification for any parenting outcomes and strata-specific CIs were wide (table 3).

10.1136/bmjgh-2023-013787.supp8 Supplementary data

10.1136/bmjgh-2023-013787.supp9 Supplementary data

10.1136/bmjgh-2023-013787.supp10 Supplementary data

**Table 3:** Effects of receiving PIM starting in pregnancy or after birth on parenting outcomes

Outcome	Started during pregnancy (n=354*)			Started after birth (n=738*)			
	β	95% CI	P value	β	95% CI	P value	Heterogeneity P values
Linear regression for mean parenting score (β)							
Parent responsiveness	−0.02	−0.29 to 0.15	0.82	0.03	−0.08 to 0.14	0.60	0.63
Parent sensitivity	0.06	−0.10 to 0.22	0.44	0.07	−0.04 to 0.18	0.23	0.95
Parent coerciveness	0.19	−0.27 to 0.65	0.42	−0.19	−0.48 to 0.09	0.19	0.53
Parent guidance	0.04	−0.04 to 0.13	0.30	0.03	−0.04 to 0.09	0.39	0.79
Logistic regression for OR							
	**OR**	**95% CI**	**P value**	**OR**	**95% CI**	**P value**	
Quality of parent–child relationship	1.35	0.84 to 2.14	0.21	0.94	0.69 to 1.26	0.67	0.71

*Early childhood development (ESS) is the effective sample size and is used in cases of full matching when matched samples are calculated via stratification and weighting. In the case of this moderation analyses, the ESS for families that started PIM during pregnancy is n=354, where there are 234 controls and 120 treated individuals. The ESS for families that started PIM after birth is n=738, where there are 270 controls and 468 treated individuals.

PIM, Primeira Infância Melhor.

Finally, moderation analyses were conducted according to length of programme involvement (<12 months vs ≥12 months; Online supplemental figure 8). Matching in both strata yielded good balance on most SMDs and VRs (online supplemental figures 9 and 10). No evidence of an effect was found for any parenting outcomes in either group (table 4).

10.1136/bmjgh-2023-013787.supp11 Supplementary data

10.1136/bmjgh-2023-013787.supp12 Supplementary data

10.1136/bmjgh-2023-013787.supp13 Supplementary data

**Table 4:** Effects of receiving PIM for >12 months or ≤ 12 months on parenting outcomes

Outcome	Caregiver receives PIM > 12 months (n=534*)			Caregiver receives PIM ≤ 12 months (n=543)		
	β	95% CI	P value	β	95% CI	P value
Linear regression for mean parenting score						
Parent responsiveness	0.04	−0.08 to 0.16	0.52	0.06	−0.06 to 0.19	0.34
Parent sensitivity	0.04	−0.10 to 0.18	0.58	0.11	−0.02 to 0.24	0.10
Parent coerciveness	0.04	−0.32 to 0.40	0.84	0.01	−0.36 to 0.38	0.94
Parent guidance	0.03	−0.04 to 0.10	0.46	−0.04	0.10 to 0.02	0.16
Logistic regression for OR estimated using via bootstrapping and CIs via BCa						
	**OR**	**95% CI**		**OR**	**95% CI**	
Quality of parent–child relationship	1.09	0.72 to 1.61		0.91	0.65 to 1.41	

*Early childhood development (ESS) is the effective sample size and is used in cases of full matching when matched samples are calculated via stratification and weighting. In the case of this moderation analyses, the ESS for caregivers who received PIM for >12 months is n=534, where there are 289 controls and 245 treated individuals. The ESS for caregivers who received PIM for ≤ 12 months is n=543, where there are 201 controls and 342 treated individuals.

BCa, Bias-corrected and accelerated bootstrap interval; PIM, Primeira Infância Melhor.

## Discussion

Our ability to achieve better outcomes for young children rests on gaining a more nuanced understanding of the mechanisms by which parenting interventions operate. This study evaluated whether a real-world, large-scale home-visiting programme targeting vulnerable families in Brazil improved parenting practices when children were age 4 years. Overall, PIM had modest positive effects on two observational measures of parent sensitive responsiveness. Effect sizes were 0.10 and 0.12, respectively. These findings are encouraging as they suggest that population-level public policies targeting at-risk caregivers in LMIC settings can improve the quality of sensitive care that children receive. Null findings on more distally related-parent outcomes—including parent guidance, coerciveness and the parent–child relationships—indicate that improvements in positive parenting were not ubiquitous.

While previous evaluations in LMIC settings have tended to report larger effects on responsive parenting practices,^13^ it is important to note that PIM’s operation as a standard-care, state-wide public policy likely contributed to its smaller effects. Further, responsive caregiving was assessed on-average 2 years post intervention. Longitudinal follow-ups often find a dilution of effects over time, particularly on parenting practices.^53^ For example, in their effectiveness evaluation of the Lady Health Worker Program in Pakistan, Yousafzai *et al* found that intervention effects on responsive caregiving decreased from large (d=0.8), immediately post intervention, to small (d=0.2) at 2-year follow-up.^54^ Similarly, in their follow-up of Programa Cuna Mas in Peru, Araujo *et al* found that effects on maternal stimulation were not sustained at 2 years.^12^

The positive effects of PIM on sensitive responsiveness observed using two different measures and two different tasks—one involving shared book-reading and the other a semistructured play activity—also suggest that caregivers who received the programme were able to adapt the responsive caregiving skills they learnt from one context to another. This finding demonstrates the generalisability of the responsiveness construct to daily care activities and is corroborated by previous studies in HICs.^55^ To the authors’ knowledge, this is the first real-world, effectiveness study in an LMIC setting to find evidence for observational adaptability of sensitive responsiveness in multiple contexts of care.

Additionally, findings from this evaluation strengthen PIM’s scientific evidence-base^24 28 56^ and empirically establish responsive caregiving as a key mechanism of change in the intervention’s logic model. They also build on the small body of parenting intervention research previously conducted in Brazil, which has been primarily limited to small-scale, researcher-designed interventions.^57–59^ An exception to this is a recent large-scale cluster RCT of Brazil’s federal home-visiting programme, CF, across 30 municipalities.^60^ At 24-month follow-up, no improvements in any parent or child outcomes were observed.^60^ CF implementation data suggests that the lack of impact at follow-up was likely due to low dosage, poor delivery and high turn-over—challenges which are pervasive in effectiveness study research. On balance, this leaves us with a mixed picture of parenting intervention research at-scale in Brazil.

There was some evidence of differential effects of PIM across the three potential moderators that were tested. First, it appears that PIM may have been more effective at improving responsive caregiving in low- income parents compared with higher income parents, though these results should be considered with caution given heterogeneity tests just above significance. Previous research indicates strong negative associations between socioeconomic risk and parent sensitive responsiveness.^61^ Parents facing economic deprivation often experience a host of other stressors, including insecure housing, diminished social networks and neighbourhood violence.^62^ Collectively, these factors may tax caregivers’ cognitive bandwidth, making it more difficult for them to engage in responsive care.^63^ Given this, PIM’s positive effect on sensitive responsiveness in the low-income parent subgroup is particularly promising.^64^ High-quality, responsive stimulation during early childhood can attenuate the effects of poverty on child development. In view of this, PIM may represent a developmentally protective pathway against poverty-related stressors via increases in responsive care. Given that low-income families in Brazil experience lower rates of access to other protective, early care services, such as daycare and preschool,^65^ home-visiting may present a unique and powerful policy lever to reach these families and increase quality early stimulation for children who need it most. Differential benefits for low-income families also provide evidence to purposefully target families living in poverty as PIM programme recipients and use family income as inclusion criteria. Explicit targeting of families where differential effectiveness has been proven is a public policy strategy which is gaining traction in the field^19 21 66^ and it is hoped that future real-world parenting intervention evaluations consider SES as a key effect modifier of programme effects on parenting. There were no significant differences between high-income and low-income groups on indicators of programme entry, programme departure or length of programme involvement, suggesting that implementation factors were unlikely to explain this finding.

There was no evidence of effect modification for either when families started PIM or for the length of time they received the programme. Null effects on parenting outcomes in the pregnancy subgroup (n=120), where we had hypothesised an effect due to previous study findings,^24^ may be explained by the small sample size and thereby limited power. It is also possible that PIM started prenatally influenced early (eg, 0–2 years) parental outcomes, but those effects waned by age 4 when current study outcomes were measured. Previous meta-analytic research on the topic of child age and programme entry remains largely inconclusive.^13 67^ A recent effectiveness study of a similar play-based home-visiting programme in Peru did not find heterogeneity of treatment effects by child age.^12^

Families who received PIM for 12 months or longer did not demonstrate differential improvements in parenting compared with families who received the programme for a shorter duration. The length of time which caregivers participated in PIM acted as a rough proxy for the number of home visits they received. It is, however, possible that families who participated in PIM for fewer than 12 months may have received a similar dose of visits to those who remained in the programme for longer. This uncertainty highlights the need for PIM to explicitly capture data on dosage. Future evaluations of PIM should also seek to assess the moderating power of other key implementation elements. These include indicators of visit fidelity, home visitor training and supervision quality—factors which have been flagged in previous qualitative studies of the programme as critical components of effective delivery of the PIM, which have yet to be addressed at a policy-level.^68–70^

One important limitation of the current study is its quasiexperimental approach to answering causal questions. The lack of randomisation of participants to treatment and control conditions, despite a robust propensity score matching approach and extensive covariate adjustment, means there may be residual confounding. If there is unmeasured, residual confounding, we would expect that to result in underestimation of the effects of PIM, given that participants are selected according to social vulnerability. There was also a lack of detailed implementation data available, which means that factors related to fidelity, participant satisfaction and home visitor preparedness, among others, may have impacted intervention delivery in ways that were not considered in the current analyses. Approximately 30% of the sample was excluded due to missingness on outcome or covariate data. Similar rates of missing data are reported in other longitudinal studies conducted in LMIC settings.^71 72^ The current study possesses notable strengths related to its evaluation of a real-world, public policy parenting intervention which was implemented and evaluated within the context of a population-based birth cohort, without any involvement or interference of the research team. Analytical strengths include the various propensity score matching approaches which were applied to the sample to ensure that high-quality matching was achieved across all 25 covariates. Finally, the use of multiple validated observational measures of parenting practices in this sample (n=1181), is an additional unique strength, one that is rare in effectiveness studies conducted in LMICs. Most have relied almost exclusively on the Home Observation for Measurement of the Environment (HOME) inventory or Family Care Indicators (MCI) to assess potential changes in parenting. Despite the popularity of these ‘brushstroke’^73^ measures, their dependence on rough counts of parenting practices likely reduces their ability to detect intervention effects, especially in real-world settings where programme effects are consistently smaller and behavioural changes more nuanced.^54 74 75^

## Conclusions

The current study demonstrates that a state-wide, standard-care home-visiting programme in Southern Brazil targeting at-risk caregivers can benefit nurturing care. We found evidence for a positive effect of PIM on two measures of responsive parenting. Improvements in these core caregiving capacities suggest that PIM may serve as one powerful policy lever to promote more secure and sensitive home environments where children can grow and flourish. Our findings also suggest that PIM may have been differentially effective at improving sensitivity for low-income families, a subgroup which future effectiveness studies should seek to explicitly target and analyse.

10.1136/bmjgh-2023-013787.supp14 Abstract translation This web only file has been produced by the BMJ Publishing Group from an electronic file supplied by the author(s) and has not been edited for content.

## Data Availability

Data are available upon reasonable request. Due to confidentiality restrictions relating to the ethics approval for this study, no identifying information about participants may be released. The dataset without identification that was used during the current study is available from the corresponding author on reasonable request.
