# Supplementary material for: Towards a better understanding of real-world home-visiting programs: a large-scale effectiveness study of parenting mechanisms in Brazil
Source: BMJ Glob Health. 2024 Feb 20;9(2):e013787. doi: 10.1136/bmjgh-2023-013787 (PMC10882332; doi:10.1136/bmjgh-2023-013787)
Supplement: Supplementary data [file bmjgh-2023-013787supp001.pdf]

BMJ Publishing Group Limited (BMJ) disclaims all liability and responsibility arising from any reliance placed on this supplemental material which has been supplied by the author(s)

Supplement Table 1: Comparison of social vulnerability covariates between families who received PIM and potential control families in the analytical sample (n=3,018)

Covariate	Families who received PIM in analytical sample (n=587)	Potential controls families in analytical sample (n=2,431)
**Maternal age at birth**, mean (sd)	26.2 (6.4)	28.3 (6.5)
**Maternal education,** mean (sd)	8.1 (3.2)	11.0 (3.8)
**Paternal education**, mean (sd)	7.0 (3.3)	10.0 (3.9)
**Family income at birth**, mean (sd)	1642 (119.6)	3475 (4447.6)
**Asset index at birth**, mean (sd)	-1.4 (1.6)	0.5 (2.5)
**Resident density at birth**, mean (sd)	3.0 (1.1)	2.5 (0.9)
**Neighborhood violence**, mean (sd)	3.4 (3.1)	2.6 (2.6)
**Child weight at birth(g)** , mean (sd)	3183 (551.3)	3210 (532.4)
**No. of prenatal consultations**, n(%)		
<6	106 (18.1)	251 (10.3)
≥6	481 (81.9)	2180 (89.7)
**Maternal depression symptoms at 3 months**, n(%)		
Yes	157 (26.7)	409 (16.8)
No	430 (73.3)	2022 (83.2)
**Gestational age**, n(%)		
<37 weeks	90 (15.3)	318 (13.1)
≥37 weeks	497 (84.7)	2113 (86.9)
**Apgar at the 5th minute**, n(%)		
<7	6 (1.0)	20 (0.8)
≥7	581 (99.0)	2411 (99.2)
**No. of kids living with mother at birth**, n(%)		
0	236 (40.2)	1336 (55.0)
1	198 (33.7)	772 (31.8)
2	90 (15.3)	220 (9.0)
3	32 (5.5)	66 (2.7)
4+	31 (5.3)	37 (1.5)
**Mother's skin color/race**, n(%)		
White	337 (57.4)	1856 (76.3)
Black	140 (23.9)	307 (12.6)
Mixed race	1 (0.2)	10 (0.4)
Asian	107 (18.2)	254 (10.4)
Indigenous	2 (0.3)	37 (1.5)
**Father's degree of pregnancy support**, n(%)		
Little support	60 (10.2)	194 (8.0)
Much support	527 (89.8)	2237 (92.0)
**Mother lives with partner at birth ,** n(%)		
Yes	505 (86.0)	2210 (90.9)
No	82 (14.0)	221 (9.1)
**Main caregiver until 3 months** , n(%)		
Mother	560 (95.4)	2369 (97.4)
Other	27 (4.6)	62 (2.6)
**Mother smoked during pregnancy**, n(%)		
Yes	133 (22.7)	289 (11.9)
No	454 (77.3)	2142 (88.1)
**Mother drank alcohol during** pregnancy, n(%)		
Yes	38 (6.5)	178 (7.3)
No	549 (93.5)	2253 (92.7)
**Maternal diabetes during pregnancy ,** n(%)		
Yes	57 (9.7)	215 (8.8)
No	530 (90.3)	2216 (91.2)
**Mother paid worked during pregnancy**, n(%)		
Yes	238 (40.5)	890 (36.6)
No	349 (59.5)	1541 (63.4)
**Mother arterial hypertension during pregnancy**, n(%)		

Healy MR, *et al. BMJ Glob Health* 2024; 9:e013787. doi: 10.1136/bmjgh-2023-013787

Yes	165 (28.1)	603 (24.8)
No	422 (71.9)	1828 (75.2)
**Pregnancy planned**, n(%)		
Yes	275 (46.8)	1303 (53.6)
No	312 (53.2)	1128 (46.4)
**Child sex**, n(%)		
Male	296 (50.4)	1258 (51.7)
Female	291 (49.6)	1173 (48.3)
**Preschool attendance**, n(%)		
No preschool attendance (0-2 years)	464 (79.0)	1507 (62.0)
Some preschool attendance (0-2 years)	103 (17.5)	599 (24.6)
Full preschool attendance (0-2 years)	20 (3.4)	325 (13.4)
