# Supplementary material for: Towards a better understanding of real-world home-visiting programs: a large-scale effectiveness study of parenting mechanisms in Brazil
Source: BMJ Glob Health. 2024 Feb 20;9(2):e013787. doi: 10.1136/bmjgh-2023-013787 (PMC10882332; doi:10.1136/bmjgh-2023-013787)
Supplement: Supplementary data [file bmjgh-2023-013787supp002.pdf]

Supplement Table 2: Parent and Family Adjustment Scales (PAFAS): Coercive parenting & Quality of the parent-child relationship subscales (Schneider et. al., 2021)

**Coercive Parenting subscale**	**Parent-child relationship subscale**
Item 1: I shout or get angry with my child when they misbehave.	Item 1: I chat/talk with my child.
Item 2: I try to make my child feel bad (e.g. guilt or shame) when they misbehave, to teach them a lesson.	Item 2: I enjoy giving my child hugs, kisses and cuddles.
Item 3: I spank (smack) my child when they misbehave.	Item 3: I am proud of my child.
Item 4: I get annoyed with my child.	Item 4: I enjoy spending time with my child.
	Item 5: I have a good relationship with my child.
