# Supplementary material for: Towards a better understanding of real-world home-visiting programs: a large-scale effectiveness study of parenting mechanisms in Brazil
Source: BMJ Glob Health. 2024 Feb 20;9(2):e013787. doi: 10.1136/bmjgh-2023-013787 (PMC10882332; doi:10.1136/bmjgh-2023-013787)
Supplement: Supplementary data [file bmjgh-2023-013787supp003.pdf]

Supplemental Table 3: Missingness rates on key covariates and outcome variables across the whole cohort, analytical sample and excluded sample.

Covariate	Whole cohort (n=4,275)	Analytical Sample (n=3,018)	Excluded Sample (n=1,257)
**Maternal age at birth**, mean (sd)	27.6 (6.6)	27.9 (6.5)	27.0 (6.9)
Missing data (n)	1	0	1
**Maternal education,** mean (sd)	10.0 (4.0)	10.4 (3.9)	9.1 (4.1)
Missing data (n)	1	0	1
**Paternal education**, mean (sd)	9.4 (4.1)	9.5 (4.0)	9.1 (4.3)
Missing data (n)	252	0	252
**Family income at birth**, mean (sd)	3057.0 (4123.5)	3119.0 (4091.1)	2869 (4216.6)
Missing data (n)	259	0	259
**Asset index at birth**, mean (sd)	0.0 (2.5)	0.1 (2.4)	-0.3 (2.6)
Missing data (n)	148	0	148
**Resident density at birth**, mean (sd)	2.7 (1.0)	2.6 (0.9)	2.7 (1.1)
Missing data (n)	172	0	172
**Neighborhood violence**, mean (sd)	2.8 (2.8)	2.8 (2.7)	2.8 (2.8)
Missing data (n)	314	0	314
**Child weight at birth(g)** , mean (sd)	3170 (564.2)	3205 (536.2)	3085 (619.2)
Missing (n)	17	0	17
**No. of prenatal consultations**, n(%)			
<6	674 (15.8)	357 (11.8)	317 (25.6)
≥6	3583 (84.2)	2661 (88.2)	922 (74.4)
Missing (n)	18	0	18
**Maternal depression symptoms at 3 months**, n(%)			
Yes	831 (20.3)	566 (18.8)	265(24.6)
No	3264 (79.7)	2452 (81.2)	812 (75.4)
Missing data (n)	180	0	180
**Gestational age**, n(%)			
<37 weeks	663 (15.5)	408 (13.5)	255 (20.3)
≥37 weeks	3612 (84.5)	2610 (86.5)	1002 (79.7)
Missing data (n)	0	0	0
**Apgar at the 5th minute**, n(%)			
<7	54 (1.3)	26 (0.9)	28 (2.3)
≥7	4206 (98.7)	2992 (99.1)	1214 (97.7)
Missing data (n)	15	0	15
**No. of kids living with mother at birth**, n(%)			
0	2174 (50.9)	1572 (52.1)	602 (48.0)
1	1341 (31.4)	970 (32.1)	371 (29.5)
2	453 (10.6)	310 (10.3)	143 (11.4)
3	169 (4.0)	98 (3.2)	71 (5.7)
4+	13 (3.2)	68 (2.3)	68 (5.4)
Missing data (n)	2	0	2
**Mother's skin color/race**, n(%)			
White	3024 (70.9)	2193 (72.7)	831 (66.5)
Black	667 (15.6)	447 (14.8)	220 (17.6)
Mixed race	551 (12.9)	361 (12.0)	190 (15.2)
Asian	16 (0.4)	11 (0.4)	5 (0.4)
Indigenous	10 (0.2)	6 (0.2)	4 (0.3)
Missing data (n)	7	0	7
**Father's degree of pregnancy support**, n(%)			
Little support	462 (11.0)	254 (8.4)	208 (17.6)
Much support	3736 (89.0)	2764 (91.6)	972 (82.4)
Missing data (n)	77	0	77
**Mother lives with partner at birth ,** n(%)			
Yes	3667 (85.8)	2715 (90.0)	952 (75.8)
No	607 (14.2)	303 (10.0)	304 (24.2)
Missing (n)	1	0	1
**Main caregiver until 3 months** , n(%)			
Mother	3941 (95.9)	2929 (97.1)	1012 (92.8)
Other	168 (4.1)	89 (2.9)	79 (7.2)
Missing (n)	166	0	166
**Mother smoked during pregnancy**, n(%)			
Yes	705 (16.5)	422 (14.0)	283 (22.6)

No	3567 (83.5)	2596 (86.0)	971 (77.4)
Missing (n)	3	0	3
**Mother drank alcohol during** pregnancy, n(%)			
Yes	315 (7.4)	216 (7.2)	99 (7.9)
No	3957 (92.6)	2802 (92.8)	1155 (92.1)
Missing (n)	3	0	3
**Maternal diabetes during pregnancy ,** n(%)			
Yes	366 (8.6)	272 (9.0)	94 (7.5)
No	3906 (91.4)	2746 (91.0)	1160 (92.5)
Missing (n)	3	0	3
**Mother paid worked during pregnancy**, n(%)			
Yes	2379 (55.7)	1779 (58.9)	656 (52.2)
No	1895 (44.3)	1239 (41.1)	600 (47.8)
Missing (n)	1	0	1
**Mother arterial hypertension during pregnancy**, n(%)			
Yes	1089 (25.5)	768 (25.4)	321 (25.6)
No	3183 (74.5)	2250 (74.6)	933 (74.4)
Missing (n)	3	0	3
**Pregnancy planned**, n(%)			
Yes	2058 (48.2)	1578 (52.3)	480 (38.2)
No	2216 (51.8)	1578 (47.7)	776 (61.8)
Missing (n)	1	0	1
**Child sex**, n(%)			
Male	2164 (50.6)	1554 (51.5)	610 (48.5)
Female	2111 (49.4)	1464 (48.5)	647 (51.5)
Missing (n)	0	0	0
**Preschool attendance**, n(%)			
No preschool attendance (0-2 years)	2615 (66.9)	1971 (65.6)	644 (72.5)
Some preschool attendance (0-2 years)	886 (22.7)	702 (23.3)	184 (20.7)
Full preschool attendance (0-2 years)	405 (10.4)	345 (11.4)	60 (6.8)
Missing (n)	369	0	369
**Any enrolment in PIM up to age** 4, n(%)			
Yes	797 (18.6)	587 (19.4)	210 (17.0)
No	3454 (81.3)	2431 (80.6)	1023 (83.0)
Missing (n)	24	0	24
**Responsive interactions**, mean (sd)	2.5 (0.8)	2.6 (0.8)	2.3 (0.8)
Missing (n)	407	0	407
**Parent sensitivity**, mean (sd)	3.6 (0.8)	3.7 (0.8)	3.4 (0.9)
Missing (n)	407	0	407
**Parent coerciveness** (PAFAS), mean (sd)	3.6 (2.1)	3.6 (2.1)	3.6 (2.2)
Missing (n)	297	0	297
**Parent guidance**, mean (sd)	0.7 (0.4)	0.7 (0.4)	0.60 (0.4)
Missing (n)	416	0	416
**Parent-child relationship** (PAFAS), n(%)			
0	2101 (52.8)	1651 (54.7)	450 (46.8)
1	1878 (47.2)	1367 (45.3)	511 (53.2)
Missing (n)	296	0	296
