# Supplementary material for: Towards a better understanding of real-world home-visiting programs: a large-scale effectiveness study of parenting mechanisms in Brazil
Source: BMJ Glob Health. 2024 Feb 20;9(2):e013787. doi: 10.1136/bmjgh-2023-013787 (PMC10882332; doi:10.1136/bmjgh-2023-013787)

Supplemental Figure 1: Love plot comparison of SMD & VR differences before vs. after propensity score matching in the full analytical sample of enrolment in PIM at any age.

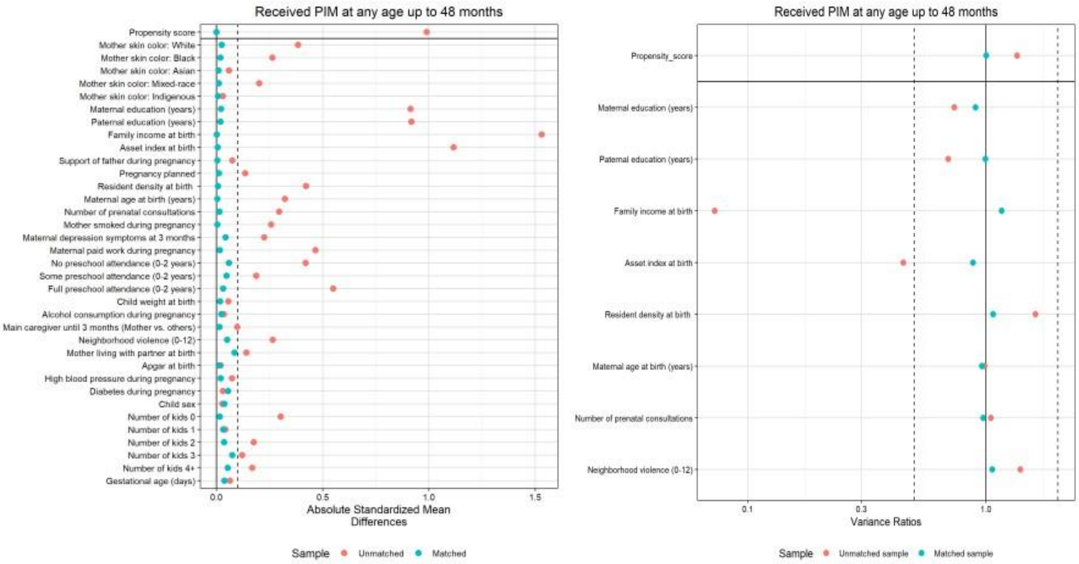

Supplement: Supplementary data [file bmjgh-2023-013787supp004.pdf]
