# Supplementary material for: Towards a better understanding of real-world home-visiting programs: a large-scale effectiveness study of parenting mechanisms in Brazil
Source: BMJ Glob Health. 2024 Feb 20;9(2):e013787. doi: 10.1136/bmjgh-2023-013787 (PMC10882332; doi:10.1136/bmjgh-2023-013787)
Supplement: Supplementary data [file bmjgh-2023-013787supp007.pdf]

Supplemental Figure 4A & 4B: Love plot comparison of SMD & VR differences before vs. after propensity score matching in the analysis of PIM on lower income caregivers.

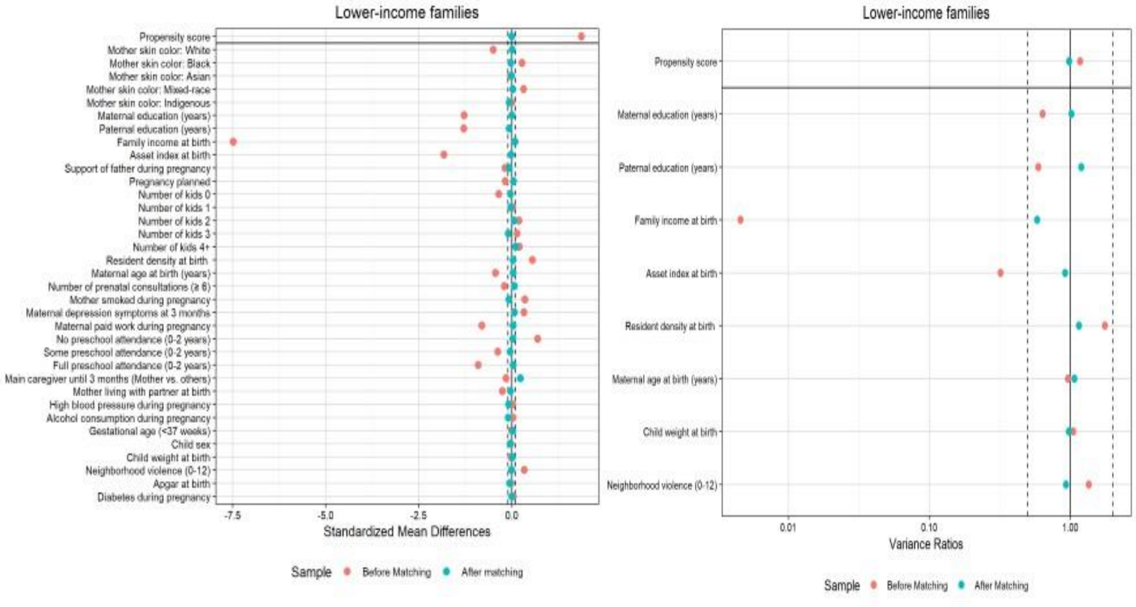

**The covariates of main caregiver up until 3 months of age and caregiver resides with 4+ children were just above 0.1 absolute SMD threshold cutoffs. As such, these covariates were added as predictors to all outcome regression model.*
