# Supplementary material for: Towards a better understanding of real-world home-visiting programs: a large-scale effectiveness study of parenting mechanisms in Brazil
Source: BMJ Glob Health. 2024 Feb 20;9(2):e013787. doi: 10.1136/bmjgh-2023-013787 (PMC10882332; doi:10.1136/bmjgh-2023-013787)

BMJ Publishing Group Limited (BMJ) disclaims all liability and responsibility arising from any reliance placed on this supplemental material which has been supplied by the author(s)

Supplemental Figure 7A & 7B: Love plot comparison of SMD & VR differences before vs. after propensity score matching in the analysis of **PIM after birth.**

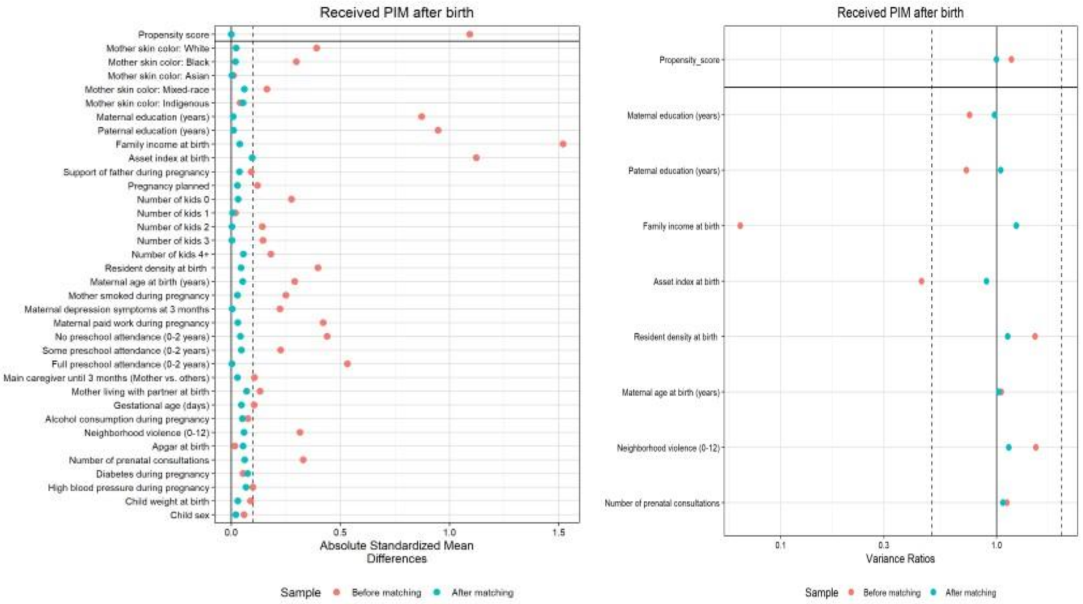

Supplement: Supplementary data [file bmjgh-2023-013787supp010.pdf]
