# Supplementary material for: Towards a better understanding of real-world home-visiting programs: a large-scale effectiveness study of parenting mechanisms in Brazil
Source: BMJ Glob Health. 2024 Feb 20;9(2):e013787. doi: 10.1136/bmjgh-2023-013787 (PMC10882332; doi:10.1136/bmjgh-2023-013787)
Supplement: Supplementary data [file bmjgh-2023-013787supp013.pdf]

<=

Supplemental Figure 10A & 10B: Love plot comparison of SMD & VR differences before vs. after propensity score matching in the analysis of **PIM on caregivers with ≥ 12 months** program involvement. (<=) (<=)

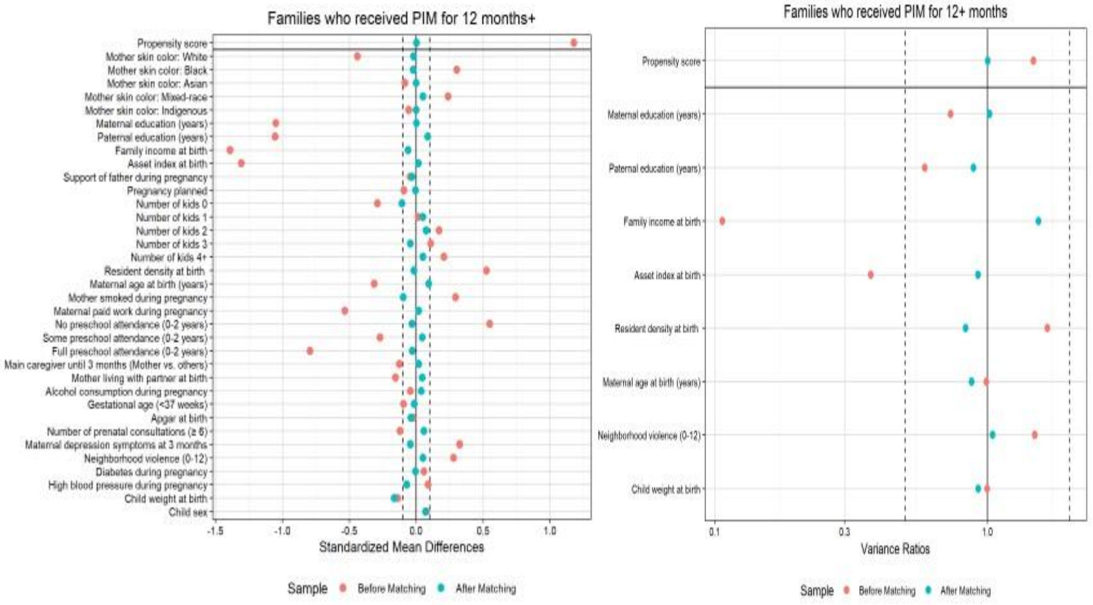

**The covariates of child weight at birth and caregiver resides with 0 children were just above 0.1 absolute SMD threshold cutoffs. As such, these covariates were added as predictors to all outcome regression models.*
