# Supplementary material for: Towards a better understanding of real-world home-visiting programs: a large-scale effectiveness study of parenting mechanisms in Brazil
Source: BMJ Glob Health. 2024 Feb 20;9(2):e013787. doi: 10.1136/bmjgh-2023-013787 (PMC10882332; doi:10.1136/bmjgh-2023-013787)
Supplement: Abstract translation [file bmjgh-2023-013787supp014.pdf]

PORTUGUESE TRANSLATION OF ABSTRACT

Introdução

A ampliação em larga escala de programas parentais para apoiar o desenvolvimento na primeira infância é pouco compreendida. Se conhece pouco acerca de como ou quando tais intervenções precoces são mais efetivas. A sustentabilidade destes programas requer um melhor entendimento dos mecanismos das intervenções em contexto de mundo real. Examinamos os efeitos sobre práticas de cuidado parental do programa de visitas domiciliares Primeira Infância Melhor (PIM), com abrangência estadual, no Brasil.

Métodos

Este estudo longitudinal, quase-experimental com pareamento por escore de propensão, utiliza dados da Coorte de Nascimentos de Pelotas de 2015. Nós pareamos crianças que receberam o PIM em qualquer idade com outras crianças da coorte que não o receberam, com base em 25 covariáveis. Sensibilidade, orientação e responsividade parentais foram avaliadas por meio de tarefas-brincadeira gravadas em vídeo. A coercitividade e o relacionamento parentais com a criança foram avaliados com uso das escalas PAFAS. Todos os desfechos parentais foram mensurados aos 4 anos de idade da criança. Análises de moderação em separado foram conduzidas para cada modificador do efeito: renda familiar, idade da criança e duração da participação no PIM.

Resultados

Do total de 4275 crianças da coorte, 797 receberam o PIM até os 4 anos de idade. 3018 crianças (70,6%) foram incluídas na amostra analítica, das quais 587 receberam o PIM e 2431 foram potenciais controles. Encontramos efeitos positivos do PIM sobre responsividade ($\beta = 0{,}08$, IC95% 0,002 a 0,16) e sensibilidade ($\beta = 0{,}10$, IC95% 0,02 a 0,19) parentais. Não foram encontrados efeitos sobre nenhum dos desfechos secundários. As análises de modificação do efeito revelaram um efeito positivo mais forte sobre sensibilidade parental para o subgrupo de mais baixa renda familiar ($\beta = 0{,}18$, IC95% 0,03 a 0,34).

Conclusão

Um programa de visitas domiciliares de abrangência estadual no Brasil melhorou aspectos do cuidado parental. Os efeitos foram mais pronunciados para famílias de mais baixa renda, sugerindo benefícios maiores do programa na medida em que melhore o foco em famílias mais vulneráveis.
